# Enhancement of Poly(Lactic Acid) Fire Retardancy Through the Incorporation of Sludge Residue as a Synergistic Additive

**DOI:** 10.3390/polym17202717

**Published:** 2025-10-10

**Authors:** Jimena de la Vega, Antonio Vázquez-López, De-Yi Wang

**Affiliations:** 1 IMDEA Materials Institute, C/Eric Kandel, 2, 28906 Madrid, Spain; jimena.vega@imdea.org; 2 Department of Materials Science, ETS Ingenieros de Caminos, Polytechnic University of Madrid, 28040 Madrid, Spain; 3 Materials Science and Engineering Area, Escuela Superior de Ciencias Experimentales y Tecnología, Universidad Rey Juan Carlos, C/Tulipán s/n, 28933 Madrid, Spain; antonio.vazquez@urjc.es

**Keywords:** sludge, waste, synergist, fire retardancy, Poly(Lactic Acid)

## Abstract

The escalating global challenge of waste production underscores the urgency for innovative waste management solutions. Sewage sludge, a byproduct derived from anaerobic digesters of wastewater treatment, was investigated as a flame-retardant synergist in Poly(Lactic Acid) (PLA). Micronized sludge was combined with ammonium polyphosphate (APP) at different ratios. The formulation containing (4:1) APP:Sludge exhibited enhanced flame retardancy compared to APP alone, achieving higher Limiting Oxygen Index (LOI) values and a V-0 rating in the UL-94 test. Cone calorimeter analysis further confirmed that the sludge contributed to reducing heat release and smoke generation. SEM–EDS analysis indicated that microcrystals, mainly composed of phosphorus and calcium oxides from APP and sludge, likely acted as protective barriers against heat transfer. In addition, filament extrusion demonstrated that sludge incorporation is compatible with 3D printing. This approach preserved structural integrity, sustainably utilized sewage sludge, and reduced reliance on commercial flame retardants. Integrating sludge as a synergist offers a promising solution for waste management and safer, more sustainable flame-retardant materials, supporting a circular economy.

## 1. Introduction

The burgeoning issue of waste production continues to be a significant global challenge. According to the World Bank, the world generates around 2.01 billion tonnes of municipal solid waste annually, increasing to a staggering 3.40 billion tonnes by 2050 while at least 33% of this waste is currently mismanaged through environmentally unsafe practices such as open dumping or burning [1]. Among these vast amounts of waste, the contribution of urban waste, especially those originating from wastewater (sludge) is considerable, with the cost of handling and disposal of waste sludge being up to 50% of the wastewater treatment cost [2]. Sludge in Wastewater Treatment Plants (WWTPs) originates from the removal of solids and organic matter during wastewater treatment, including primary and secondary sludge. In particular, this sewage sludge produced in WWTPs is estimated to be around 45 million tonnes of dry solids annually in the world [3]. This large-scale generation of sludge poses various environmental and management issues since it tends to accumulate heavy metals, microplastics, and trace organic compounds with low biodegradability, as well as potentially pathogenic entities (such as viruses and bacteria) [4]. The excess sludge not only puts pressure on the available resources for waste disposal but also contributes to environmental pollution if not properly treated and disposed of.

Anaerobic digestion of the sewage sludge is often implemented in WWTPs after primary and secondary wastewater treatment, which mitigates the degradation and odour of sludge, reduces harmful microbial content, and diminishes biosolid volumes, facilitating biogas production [5]. The remanent solid residue after anaerobic digestion, known as *digestate*, is commonly dewatered before final disposal. The resulting digestate, rich in nutrients like nitrogen and phosphorus from sources such as human waste and certain detergents, has the potential to be a fertilizer component in agriculture. However, as pathogen reduction varies and harmful bacterial regrowth is not always prevented, some authorities are imposing stricter rules on its use [6]. This issue, coupled with the fact that modern systems often display flaws in biogas extraction efficiency from anaerobic digestion, underscores the urgency to explore innovative alternatives for the usage of this digestate.

In the realm of the circular economy, one avenue for waste management involves repurposing municipal sewage sludge as a resource across various industries, such as in clay production [7]. In recent times, sewage sludge has been disposed of in landfills, stored in large ponds, sun-dried in arid climates, or even dumped into the oceans. For many years, land application was the primary disposal method within the European Union (EU). However, dwindling public acceptance and stricter regulations have emerged as significant constraints on this practice [8]. Consequently, over the past two decades, researchers have turned their attention towards reusing and recycling sewage sludge to foster a more sustainable environment. One key objective is to increase the amount of sewage sludge treated via thermal methods, such as incineration [8]. While incineration reduces the volume of municipal sewage sludge and yields energy, it is not a definitive solution, as it generates bottom and fly ashes that necessitate subsequent disposal [9]. Therefore, the growing need for environmentally friendly and sustainable waste management solutions has steered focus toward innovative uses of waste materials. In this sense, sludge is emerging as a valuable resource with untapped potential.

In addition, the world is witnessing a growing demand for materials with flame-retardant properties due to increasing safety standards across several industries [10].

Sewage sludge is a heterogeneous by-product of wastewater treatment, and its composition strongly depends on the treatment stage and conditions. Sludge collected from the primary and secondary treatment steps of a WWTP is rich in organic matter, microbial biomass, and extracellular polymeric substances (EPS). When subjected to anaerobic digestion, however, this material is converted into *digestate*, a stabilized by-product with a reduced EPS content, a higher proportion of mineral matter (P, Ca, Mg, Fe), and a significant fraction of low-molecular-weight organics. This compositional distinction is essential, since digestate differs substantially from activated sludge or aerobic granular sludge, which are biologically active and EPS-rich. In recent years, several studies have highlighted the potential of sludge-derived EPS and biopolymers as flame-retardant additives. For instance, EPS recovered from aerobic granular sludge has been shown to reduce the flammability of synthetic polymers by promoting char formation [11]. Similarly, flocculant sludge has been investigated as a source of extractable biomaterials with potential fire-protection applications [12]. The inorganic elements present in sewage sludge, such as metallic oxides, can also contribute to flame retardancy [13], and a recent study demonstrated the effect of sludge-based suppression materials on the explosive flame characteristics of aluminum dust [14]. Kim et al. [15] recovered EPS and improved the fire performance of polypropylene (PP), as EPS–cellulose fibers enhanced char formation. Others have reported that flax fibers increased their flame retardancy when combined with activated sludge and aerobic granular sludge [2]. These studies clearly demonstrate that EPS-rich sludge fractions and other sludge-derived biomaterials represent a promising pathway for the development of bio-based flame-retardants. However, they primarily address aerobic sludge matrices where EPS extraction is feasible, and thus their findings cannot be directly extrapolated to anaerobically digested sludge. In contrast, the present work focuses on digestate, a residue with markedly different physicochemical properties such as stabilized organic matter, lower EPS content, and higher levels of phosphorus and other mineral species that may act synergistically in flame-retardant mechanisms. To the best of our knowledge, the direct use of digestate as a synergistic flame-retardant additive in PLA composites has not yet been explored.

Poly(Lactic Acid) (PLA), a bio-based degradable polymer, is inherently flammable with a limited oxygen index of 18–20% [16], which comprises a major challenge that restricts its broader applications [17,18]. Thus, PLA was combined with halogen-free fire retardants such as ammonium polyphosphate (APP) to boost its fire retardancy. However, the fire retardancy of APP typically requires high dosages (15–30%) [17] due to its low efficiency in char formation. As a result, additives were often combined with APP, allowing for a reduction in its content while enhancing fire retardancy through a synergistic approach [19,20,21]. In this context, bio-based flame-retardants have garnered significant interest as a solution to enhance the flame resistance of PLA materials [22]. Recently, it has been demonstrated that microalgae obtained from wastewater and enriched with phosphorus (P-Algae) enhanced the LOI (Limited Oxygen Index), increasing it up to 25.1% [23]. Another relevant study has shown that the integration of sludge fiber waste and kraft lignin powder into a PLA matrix improved both the mechanical and flame-retardant properties of the composites [24]. However, no report has yet employed digestate from the anaerobic digestion of sewage sludge as a flame-retardant synergist in PLA, aiming to decrease the content of common fire retardants such as APP while providing a valorization route for this underutilized waste stream.

In this context, the aim of this study was to investigate the potential of sludge derived from the anaerobic digestion of a wastewater treatment plant (WWTP), commonly referred to as *digestate*, as a synergistic flame-retardant additive. Firstly, a preliminary analysis was carried out to determine the sludge composition and to obtain a uniform additive by milling it to a homogeneous particle size. Subsequently, poly(lactic acid) (PLA), a biodegradable thermoplastic, was used as the polymer matrix, incorporating *digestate* (hereafter referred to as *sludge* for simplicity) as a flame-retardant synergist in combination with ammonium polyphosphate (APP). Formulations were prepared varying total additive concentrations (6–8 wt%) and different APP:Sludge ratios (4:1 and 3:2).

## 2. Materials and Methods

### 2.1. Materials

Dehydrated sludge (digestate) from the anaerobic digestion process with a moisture content of 80%, was collected from a Wastewater Treatment Plant (WWTP, Aranjuez, Spain). NatureWorks Ingeo PLA 4043D (Naarden, The Netherlands) was employed as the polymeric matrix. Ammonium polyphosphate (FR CROS 484, Phase II) was supplied from Budenheim Ibérica S.L.U. (Zaragoza, Spain).

### 2.2. Sample Preparation

#### 2.2.1. Sludge Treatment

The sludge was produced in an anaerobic digester operating at 37 °C for 30 days, being a mixture of primary, biological, and secondary sludges subjected to flotation. Within this process, anaerobic bacteria break down organic matter, generating biogas. After harnessing the biogas for renewable energy, the residual hydrated digestate (sludge) was chosen as our primary raw material.

**Figure 1:**
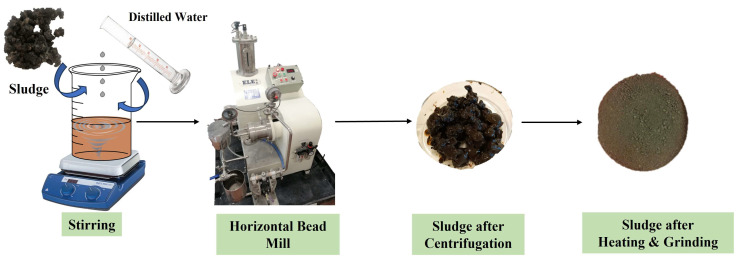
Preparation scheme of the sludge treatment to produce micronized sludge.

The sludge from the treatment plant was processed as depicted in Figure 1, through particle size reduction. For this purpose, 60 g of the sludge were dispersed in distilled water and stirred in a 2 L beaker for 30 min to ensure complete dispersion of the sludge. Then, the solution was fed into the bead mill. Various tests were conducted with different exposure times and motor speeds to achieve the maximum particle size reduction. After each time and at each speed, a small sample of the sludge was extracted, and its particle size D_50_ was quantified. Once the sludge particle size was reduced, it was exposed to convection drying in an oven at 80 °C for 12 h. The dried product was then subjected to a final milling process using a mechanical grinder, finally obtaining powdered material.

#### 2.2.2. Preparation of PLA Composites

**Figure 2:**
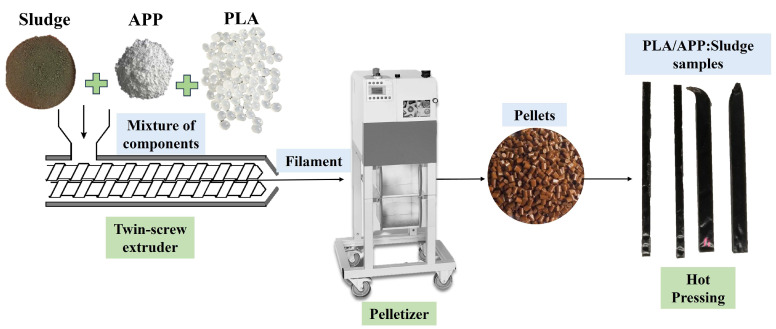
Preparation scheme of the PLA composites.

Firstly, PLA, APP, and micronized sludge were dried in a conventional oven at 85 °C for 12 h. Then, the PLA composites were uniformly prepared using identical procedures and according to the formulations in Table 1. The samples were produced by incorporating the fillers into the polymer matrix via hot-melt extrusion, utilizing a twin-screw extruder (Brabender KETSE 20/40, Duisburg, Germany), with a processing temperature of 180 °C and a rotor speed of 80 rpm. Then, the filament was immediately pelletized. Subsequently, the required test specimens were prepared for further testing using a hot plate press (Fontijne, Rotterdam, The Netherlands). The manufacturing process of the PLA/APP-Sludge composites loaded at different ratios was summarized in Figure 2.

### 2.3. Characterization

Sludge particle diameter was reduced in a two-step process using a horizontal wet bead mill (ELE, Shanghai Mechanical and Electrical Equipment Co., Ltd., Shanghai, China) and a mechanical grinder (IKA M20, Staufen, Germany). Particle size analysis was conducted using a particle size analyzer (Bettersizer ST-9300 ST, Changsha, China).

Thermal stability of PLA/APP:Sludge composites was determined by thermo-gravimetric analysis TGA (Q50, TA Universal Analysis, New Castle, DE, USA) under N_2_ atmosphere from room temperature to 800 °C at a speed rate of 10 °C/min. Thermogravimetric Analysis coupled with Fourier Transform Infrared Spectroscopy (TGA-FTIR) was employed to investigate the decomposed volatiles. Samples of 15 ± 0.2 mg underwent heating from room temperature to 800 °C at a rate of 10 °C/min in a nitrogen atmosphere and were subsequently analyzed via FTIR with a resolution of 4 cm^−1^, with a range from 4000 cm^−1^ to 400 cm^−1^.

The crystalline structure was examined by X-ray diffraction (XRD), employing a Philips PANalytical X’Pert Pro system (Almelo, The Netherlands) with Cu Kα radiation.

Differential Scanning Calorimetry DSC tests (Q200, TA Universal Analysis, New Castle, DE, USA) were conducted under a nitrogen atmosphere with a heating rate of 10 °C/min and a temperature range from 0 °C to 200 °C. To determine the enthalpies of crystallization and melting, the areas under the DSC cooling and heating curves were integrated. The degree of crystallinity was then calculated using Equation (1):

(1) $$X_{c}\left(\%\right)=\frac{{∆H}_{m}-{∆H}_{cc}}{{{∆H}_{m}}^{0}w_{p}}\times 100$$

where ΔHm is the melting entalphy, ΔHcc is cold crystallization enthalpy, ΔHcc^0^ is melting enthalpy of 100% crystalline polymer (93 J/g for PLA [25]) and *w_p_* is the mass fraction of the polymer.

Sample visualization, char observation and elemental mapping were performed using a Scanning Electron Microscope (FE-SEM, Thermo Fisher Scientific Apreo 2S, Waltham, MA, USA) equipped with Energy Dispersive X-ray Spectroscopy (EDX). Samples were gold-coated for SEM observation with fractured surface samples pre-treated by immersion in liquid nitrogen before sputtering.

The Limiting Oxygen Index (LOI) (FTT Ltd., East Grinstead, UK) was obtained according to the ASTM D2863-22 standard [26], using samples with dimensions of 120 × 6.5 × 3.2 mm^3^. The results were averaged from five parallel specimens.

The UL-94 vertical burning test (FTT Ltd., UK) was conducted using a vertical fire chamber with five averaged specimens measuring 120 × 13 × 3.2 mm^3^, following the ASTM D3801-20 standard [27].

The forced flame combustion characteristics of both PLA and its composites were evaluated using a cone calorimeter (FTT Ltd., UK), in accordance with the ISO 5660-1 standard [28]. Specimens measuring 100 × 100 × 3 mm^3^ were positioned horizontally beneath the cone with a 25 mm spacing between the conical heater and the specimen. The cone heat flux was set at 50 kW/m^2^.

Residual char after cone calorimeter tests was evaluated by FTIR (ThermoFisher Scientific) and Raman Spectroscopy (Renishaw inVia, RENISHAW Ltd., Wotton-under-Edge, UK).

Rheological tests were performed using a rheometer (Anton Paar MCR 702e Multidrive, Graz, Austria) at a constant frequency of 1Hz with a 2% oscillation.

Tensile properties of both PLA and its composites were performed in accordance with ASTM D638-14 standard [29] using an Instron 3384 testing machine (Norwood, MA, USA), where a 30 mm gauge length, a tensile speed of 2 mm/min, and a 2 kN load cell were employed. The chord tensile moduli were measured within the 0.05 to 0.25% strain range. Samples were hot-pressed and shaped in a dog-bone style with specific dimensions (2 ± 0.2 mm thickness, 5 ± 0.1 mm width in the narrow section, and an overall length of 57 ± 2 mm).

The contact angle was measured using a Drop Shape Analyzer (DSA25S, KRUSS, Hamburg, Germany).

For filament manufacturing of 3D printing materials, a precision extruder (3Devo 450, Filament Maker, Utrecht, The Netherlands) was utilized, followed by a 3D printer (Ultimaker S5, adapted for 2.85 mm filament diameter).

## 3. Results and Discussion

### 3.1. Characterization of the Treated Sludge

#### 3.1.1. Particle Size Analysis

**Figure 3:**
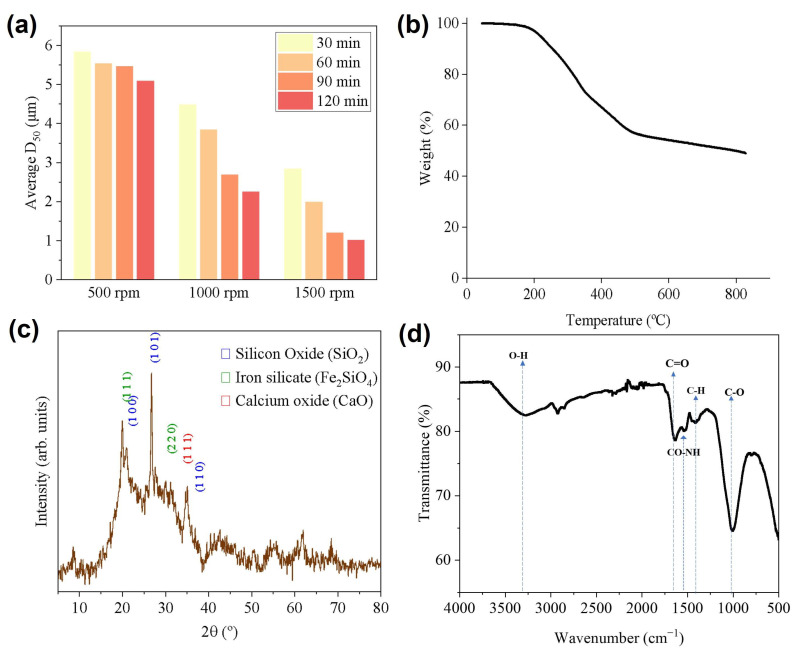
(**a**) Particle size analysis, (**b**) TGA curve, (**c**) XRD spectra and (**d**) FTIR of the micronized sludge.

Different exposure times and motor speeds used in the horizontal bead mill were tested to obtain the optimal reduction in particle size. Particle size of either additive such as APP [30] or mineral particles might influence the fire retardancy effect. Different milling speeds were evaluated from 500 rpm to 1500 rpm, as shown in Figure 3a and Table S1. The sludge from the WWTP (D_50_: 56.7 µm) exhibited a clear trend, where increasing the milling time progressively reduced the average particle size. Additionally, a significant reduction in particle size was achieved by increasing the milling speed. Ultimately, at 90 min of milling and 1500 rpm, a particle size of D_50_: 1.2 µm was achieved (Figure 3a). This condition was identified as the optimal choice from the perspective of manufacturing efficiency and particle dispersion. The micronized sludge with this average particle size was employed in the following sections.

#### 3.1.2. Thermogravimetric Analysis (TGA)

Further information regarding the composition and, mostly, the thermal stability of the sludge, was obtained by means of thermogravimetric analysis (TGA). Several studies were focused on the assisted pyrolysis obtained via TGA as a promising way for energy production from solid waste [31].

As illustrated in Figure 3b, the thermogravimetric analysis revealed three distinct decomposition stages. The first stage, occurring between 200 and 300 °C, corresponded to the degradation of readily biodegradable organic matter and semi-volatile compounds. The second stage, spanning 300–450 °C, was attributed to the thermal decomposition of hydrocarbons and alcohols. In the final stage, above 450 °C, the degradation of more thermally stable compounds, such as cellulose and other recalcitrant organics, became predominant [32]. This combustion behavior was similar to previous reports with the aerobic sludges studied in the literature [33].

#### 3.1.3. XRD

XRD was employed to identify the crystalline species that might be present in the sludge. From the XRD spectrum shown in Figure 3c, it can be concluded that both crystalline and amorphous phases were present, due to the broad and noisy spectrum with very narrow peaks which corresponded to the crystalline diffractions. The XRD spectrum of the micronized sludge revealed several peaks corresponding to different crystalline phases. Specifically, SiO_2_ was detected at 2θ angles of 20.8°, 26.6°, and 36.5° (ICDD, 00-005-0490). The presence of SiO_2_ was attributed to the natural occurrence of silicon dioxide, which is commonly found in various minerals and geological formations. Additionally, peaks corresponding to iron silicate were observed at 2θ angles of 19.8° and 32.7° (ICDD 01-084-7750). The presence of iron silicate was due to the incorporation of iron compounds in the biological water treatment in a WWTP to reduce the levels of ammonia in the water, as previously mentioned. Furthermore, calcium oxide was detected at a 2θ angle of 34.9° (ICDD 01-077-7243). The presence of calcium oxide suggested that calcium-containing minerals or compounds, such as calcium carbonate or calcium hydroxide, may be present in the sludge. These minerals could be derived from the raw materials used in wastewater treatment processes or from environmental interactions. It is important to note that the identification of these crystalline phases did not preclude the possible presence of other secondary phases or compounds exhibiting similar crystallographic diffraction patterns.

#### 3.1.4. FTIR Analysis

Further information provided by the FTIR spectra of the micronized sludge was presented in Figure 3d, where numerous absorption peaks are evident in the spectrogram due to the complex composition of sewage sludge, as documented in references [34,35,36]. The original sludge spectrum exhibited a broad and intense absorption band at 3300 cm^−1^, resulting from O-H stretching vibrations, indicating the presence of water. Bands at approximately 2924 cm^−1^ and 2852 cm^−1^ may be attributed to symmetric and asymmetric stretching vibration absorption peaks of C-H, suggesting the existence of aliphatic chains. The occurrence of C=O stretching vibrations at 1642 cm^−1^ aligned with the presence of acids and aldehydes. The absorption peak at 1546 cm^−1^ was indicative of amides (-CONH-). C-H stretching vibrations between 1393 and 1401 cm^−1^ indicated the presence of CH_2_ and CH_3_ groups, characteristic of an alkane group and implying a significant quantity of fat hydrocarbon organic matter in the sewage sludge. A robust band around 1000 cm^−1^ could be attributed to the stretching vibrations of C-O groups, corresponding to the oxidizing nature of substances such as ether, ester, or alcohol.

#### 3.1.5. SEM-EDX

**Figure 4:**
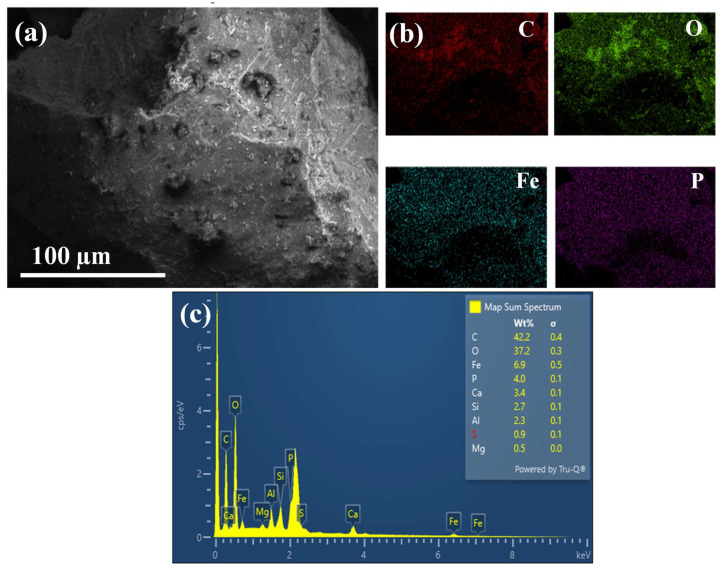
(**a**) SEM micrograph of conglomerated micronized sludge; (**b**) EDX Mapping showing Carbon (C), Oxygen (O), Iron (Fe) and Phosphorus (P); (**c**) EDX spectra.

In order to study the morphology of the sludge particles as well as their composition, scanning electron microscopy (SEM) was employed to visualize sludge conglomeration. Sludge was composed of agglomerated particles of around 150 µm (Figure 4a), which were composed primarily of carbon and oxygen (as observed in the Energy Dispersive X-ray (EDX) mapping presented in Figure 4b,c, typical of organic products from wastewater treatment plant residues. Additionally, iron presence was identified, which suggests the presence of iron-rich compounds, originating from the addition of ferric chloride during the biological treatment in the WWTP. Ferric chloride contributed to the reduction in ammonium levels in treated water by promoting sedimentation processes, as evidenced by its presence in the residual sludge generated during this stage [37,38]. Phosphorus, calcium, silicon, and aluminum were also detected. These elements could enter the purification system due to the nature of wastewater, which may contain traces of minerals and sediments present in the surrounding environment.

### 3.2. PLA Composites

#### 3.2.1. TGA

**Figure 5:**
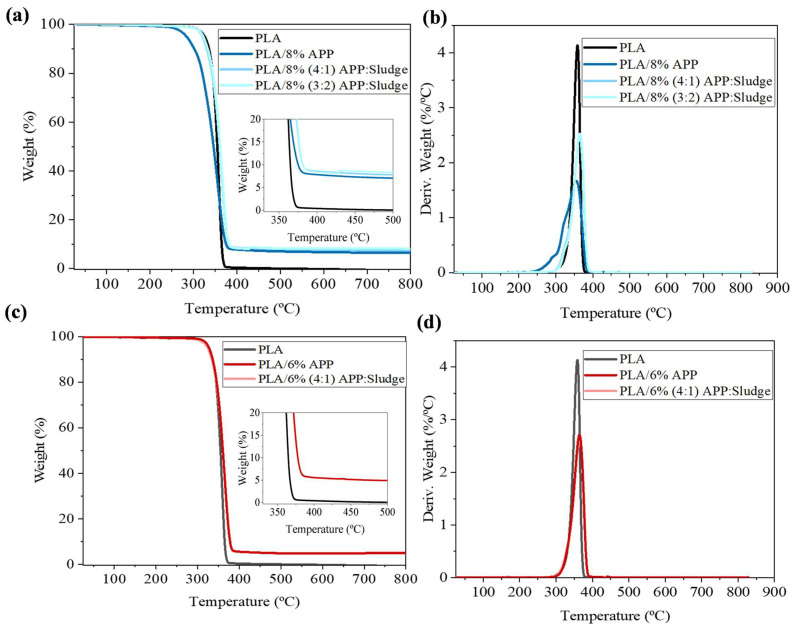
(**a**) TGA and (**b**) DTG curves of PLA and PLA composites with 8 wt% loading. The inset in (**a**) showed the region of maximum weight loss. (**c**) TGA and (**d**) DTG curves of PLA and PLA composites with 6 wt% loading. The inset in (**c**) showed the region of maximum weight loss.

TGA tests were performed to evaluate the impact of APP and sludge on the thermal stability and degradation process of PLA. Figure 5 illustrated the TGA and DTG curves of PLA and its composites of 8 and 6% loading, respectively, while Table S2 summarized decomposition parameters such as T_5%_ (temperature at 5% mass loss), T_max_ (maximum mass loss temperature), and Y_c_ (char residue yield). In a nitrogen environment, a singular primary thermal degradation step was observed for both PLA and its composites. In the case of 8% loading (Figure 5a,b) when 8%APP was added, the degradation of APP, (particularly evident at lower temperatures around 280 °C) influenced the temperature at which PLA experienced its maximum weight loss during the second stage, shifting it to lower temperatures. With the addition of sludge in both ratios of 3:2 and 4:1 APP:Sludge, maximum decomposition temperatures were increased. With respect to the char yields at 700 °C for PLA/8%APP, PLA/8% (3:2) and (4:1) APP: Sludge were recorded as 6.6%, 8.2%, and 7.6%, respectively. This observation highlighted an increase in the residual yield of PLA due to the addition of APP and sludge as a synergist. The phenomenon resulted from the thermal decomposition of APP (Figure S1), as discussed in the SI file and supported by previous works [21,39], following the generation of free acidic hydroxyl groups, ultraphosphate, and polyphosphoric acid. These compounds catalyzed the dehydration reaction of PLA, yielding char residues [40]. The presence of sludge further catalyzed and accelerated this process, consequently enhancing the yield of residual char. In this sense, it was demonstrated that metal ions facilitated the bonding of ammonium polyphosphate (APP) [41]. In this scenario, the metal compounds present in the sludge could engage in the interaction of individual phosphate groups, creating a link between two separate APP chains. This was demonstrated by the higher maximum decomposition temperature observed in PLA/8% (3:2) and (4:1) APP:Sludge. For 6% loading (Figure 5c,d), no significant changes are observed in the various decomposition parameters when adding APP or APP with sludge.

#### 3.2.2. Differential Scanning Calorimetry (DSC)

**Figure 6:**
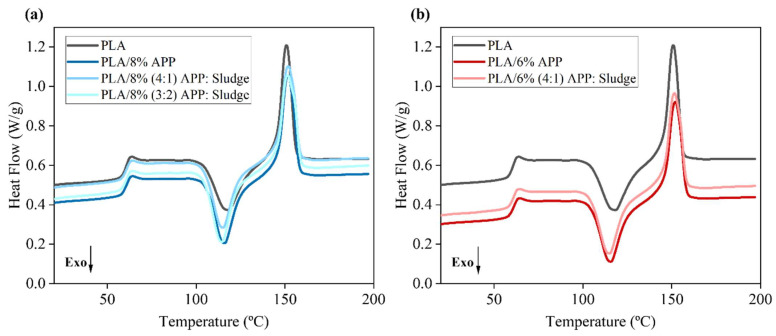
DSC curves of PLA (**a**) 8% loading and (**b**) 6% loading.

The thermal behavior and crystallization kinetics of neat PLA and its composites were investigated via DSC, as summarized in Table 2. The secondary heating curves for both PLA and its composites were shown in Figure 6.

The glass transition temperature (Tg) remained relatively stable across all formulations (63.2–63.7 °C), suggesting that the incorporation of ammonium polyphosphate (APP) and sludge-based additives did not significantly impact the segmental mobility of the PLA amorphous regions. Despite minor variations in melting temperature (Tm) (ranging from 150.9 to 152.1 °C), all samples preserved the characteristic melting profile of PLA, indicating that the crystalline lattice was not significantly altered by the additives. These results aligned with various studies where PLA composites incorporating nano-oxides (e.g., nano-silica) or biomass showed variations of less than 1 °C in Tg and Tm, indicating that the crystalline structure of PLA remained largely unaltered [25,42]. However, a moderate reduction in the cold crystallization temperature (Tc) was observed upon incorporation of APP and sludge, with the most pronounced decrease found in the PLA/8% (3:2) APP:Sludge composite (114.6 °C compared to 118.2 °C in neat PLA). This indicated a clear nucleating effect of the sludge-based filler, which facilitated earlier onset of crystallization by lowering the energy barrier for nucleation. This phenomenon has been similarly reported in systems containing mineral fillers [25,43]. The degree of crystallinity (Xc) increased in samples containing sludge, reaching up to 2.7% in the PLA/6% (4:1) APP:Sludge system, higher than in neat PLA (1.7%) or PLA/APP composites alone (1.0–1.6%). This supported the hypothesis that sludge acts as a heterogeneous nucleating agent, promoting recrystallization during the heating cycle. These results were consistent with previous studies showing enhanced crystallinity in PLA composites with inorganic nucleating agents such as calcium carbonate [44,45]. The low crystallinity of the PLA-based composites was mainly attributed to the amorphous nature of the pure polymer (PLA 4043D), which was designed for 3D printing and limited crystal formation due to rapid cooling.

#### 3.2.3. Rheology Tests

**Figure 7:**
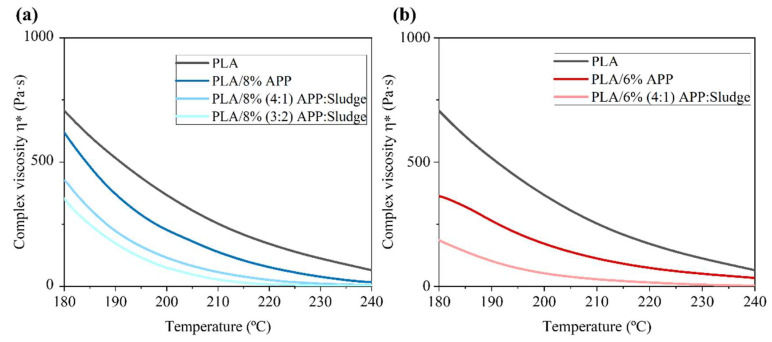
(**a**,**b**) Dependence of viscosity (η*) of the PLA with APP and sludge blends.

Rheology tests were conducted to examine the viscoelastic properties of PLA composites, the structure-property relationships in polymer blends, and the impact of sludge on PLA extrusion [46]. In order to clarify the interaction mechanism of APP-Sludge mixture in PLA, the viscosity of 8% and 6% loading of both additives in PLA blends was studied as a function of temperature. The flow behaviour of the different systems compared to the pure PLA was shown in Figure 7. The complex viscosity of the pure PLA exhibited the highest value (708 Pa·s) at the starting melting point of PLA4043D, as evidenced in other reported publications [47,48]. For samples containing sludge, lower viscosity values were observed compared to those with only APP. Several studies have demonstrated that sewage sludge could behave as a Non-Newtonian fluid; in particular, as the temperature increased, anaerobically digested sludge exhibited a gradual rise in fluidity [49,50].

The observed reduction in complex viscosity when anaerobically digested sewage sludge was present is consistent with a plasticizing or lubricating effect of components within the sludge. Sewage sludge is a complex matrix that can contain a variety of low-molecular-weight organic compounds, such as free fatty acids, mono- and diglycerides, short-chain organic acids (as detected in the sludge FTIR in Figure 3d), as well as surfactants and additives associated with microplastic contamination, including plasticizers, stabilizers, and flame retardants [51]. These soluble and extractable species can partition into the polymer melt, increasing free volume and reducing intermolecular cohesion, which lowers melt viscosity and enhances chain mobility. Fatty-acid-based and other small organic additives are known to act as effective plasticizers in PLA and other biopolymers. Additionally, surface-active compounds present in sludge may reduce interfacial friction between solid particles and the polymer matrix, yielding a more readily flowing melt [52]. Sewage sludge rheology is significantly affected by physical-chemical factors such as Total Suspended Solids (TSS) concentration, treatment methods, temperature, pH, as well as by digestion or hydrolysis processes [50]. These characteristics collectively suggested that sludge may act as a plasticizer, enhancing polymer chain mobility and reducing intermolecular interactions, which led to a less viscous material.

#### 3.2.4. SEM Cross-Section Analysis

**Figure 8:**
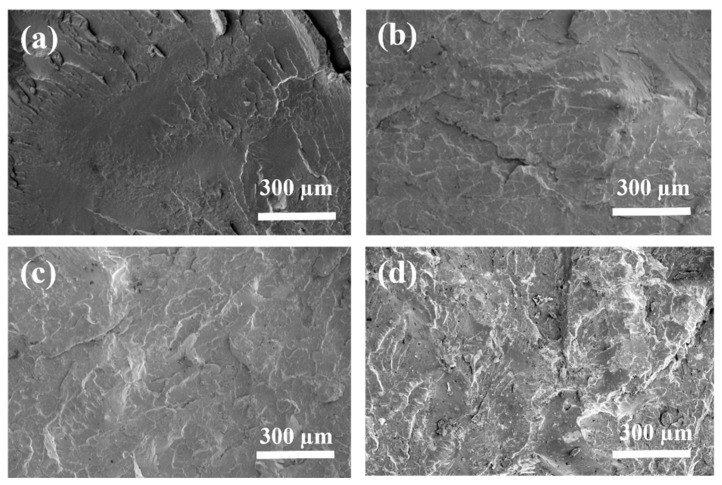
SEM cross-section of PLA composites: (**a**) PLA, (**b**) PLA/8%APP, (**c**) PLA/8% (4:1) APP:Sludge and (**d**) PLA/8% (3:2) APP:Sludge.

SEM cross-section images were obtained to investigate the dispersion of additives within the polymer matrix (Figure 8). Upon examining the cross-sections of various specimens, distinct characteristics were observed. PLA exhibited a smooth and continuous fractured surface (Figure 8a). Upon introducing 8% of APP into the polymer matrix, the composite displayed a well-dispersed filler within the PLA matrix (Figure 8b). A similar observation was done for the addition of sludge in a 4:1 ratio APP:Sludge, showing uniformity with various scattered spots corresponding to APP and sludge particles (Figure 8c). Finally, with an increased sludge content in the matrix, the fracture surface appeared rougher compared to other formulations, with a higher number of microparticles (Figure 8d). Nevertheless, the overall dispersion of fillers remained uniform.

### 3.3. Fire Retardancy

#### 3.3.1. LOI and UL94 Tests

**Figure 9:**
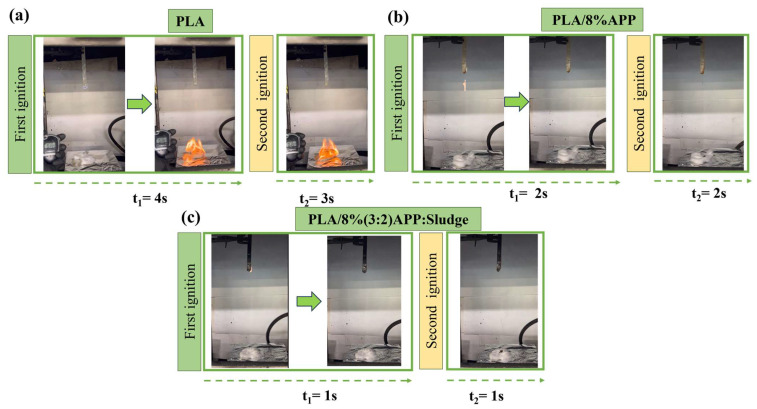
UL-94 of (**a**) pure PLA, (**b**) PLA/8%APP and (**c**) PLA/8% (3:2) APP:Sludge.

The fire safety of both PLA and its composites was first evaluated by performing LOI tests (Table 3). The LOI of pristine PLA was 22.8%, as presented in Table 3, agreeing with previous reports [17,23]. After the addition of 8% and 6% APP, the LOI value notably increased to 31.4%. APP acted as a flame retardant by releasing phosphoric acid and ammonia during combustion, which catalyzed dehydration reactions and promoted char formation [19,20,21]. Conversely, when maintaining a total additive loading of 6 wt% and 8 wt%, and incorporating 1.6 wt% and 1.2 wt% of sludge corresponding to APP:Sludge ratios of 3:2 and 4:1, respectively, the limiting oxygen index (LOI) increased to 33%.This implied that the presence of sludge contributed additively to enhancing the LOI value. The metallic oxides contained in the sludge contributed to the development of a stable char layer that effectively inhibited heat transfer and oxygen diffusion to the combustible substrate [14,46,53,54]. These results highlighted the potential role of sludge as a synergistic component, effectively enhancing the flame-retardant performance of the PLA/APP composite system. According to the UL-94 vertical burning tests (Table 3, Figure 9, and Supplementary Videos S1–S3), pure PLA burned rapidly upon ignition. It exhibited pronounced melt dripping, which ignited the cotton indicator. As a result, the material was classified as V-2 in the UL-94 test.With the incorporation of APP, a V-0 rating was achieved at a minimum loading of 6 wt%. This aligned with previous studies indicating that at least 6 wt% of APP was required for PLA composites to meet V-0 classification when used alone [55]. When APP was partially replaced with sludge at ratios of 3:2 and 4:1 (APP:Sludge), maintaining a total loading of 8 wt%, the composites also achieved a V-0 rating. This suggested that sludge contributed synergistically to the flame-retardant performance, enabling a reduction in APP content while preserving self-extinguishing behavior. The results (Figure 4c) indicated that the sludge contained significant amounts of carbon (42.2 wt%) and oxygen (37.2 wt%), consistent with its organic and mineral matrix. Importantly, phosphorus was detected at 4.0 wt%, along with smaller amounts of calcium (3.4 wt%), silicon (2.7 wt%), aluminum (2.3 wt%), iron (6.9 wt%), sulfur (0.9 wt%), and magnesium (0.5 wt%). The phosphorus content in particular is relevant to flame-retardant behavior. Phosphorus compounds are well-known for promoting char formation and inhibiting combustion in polymer matrices, acting synergistically with ammonium polyphosphate (APP). The observed V-0 rating for composites where APP was partially replaced with sludge (ratios 3:2 and 4:1, maintaining a total loading of 8 wt%) can therefore be rationalized by the presence of phosphorus and other mineral elements in the sludge, which contributed to condensed-phase flame retardancy. Calcium and phosphorus salts found in the char further enhanced char stability. Overall, the SEM-EDS data support the hypothesis that the sludge acts as a synergistic flame-retardant additive, allowing partial substitution of APP without compromising self-extinguishing behavior. This quantitative elemental analysis provides a more mechanistic understanding of the observed flame-retardant performance.

To summarize, the formulations that did not achieve V-0 rating were pristine PLA, PLA/5%APP and PLA/6% (3:2) APP:Sludge, which means that: (a) at least a content higher than 5% of loading was necessary to achieve V-0 rating and (2) that the minimum content of 6% was necessary, but with a ratio of 3:2 APP:Sludge was insufficient. It is necessary either to increase the APP:Sluge ratio with APP (4:1) or increase the whole content (up to 8% loading, either (4:1) or (3:2) APP:Sludge).

#### 3.3.2. Cone Calorimeter Tests

Following the previous results, it can be concluded that at least 6% loading is needed to achieve good flame retardancy, and the minimum ratio APP: Sludge needed is 4:1. Therefore, cone calorimeter tests were conducted on the formulation with 6 wt% total additive loading. This sample showed the lowest APP: Sludge ratio that achieved both a UL-94 V-0 rating and an LOI of 33%. It was selected for detailed analysis due to its optimal flame-retardant performance at minimal additive content.

**Figure 10:**
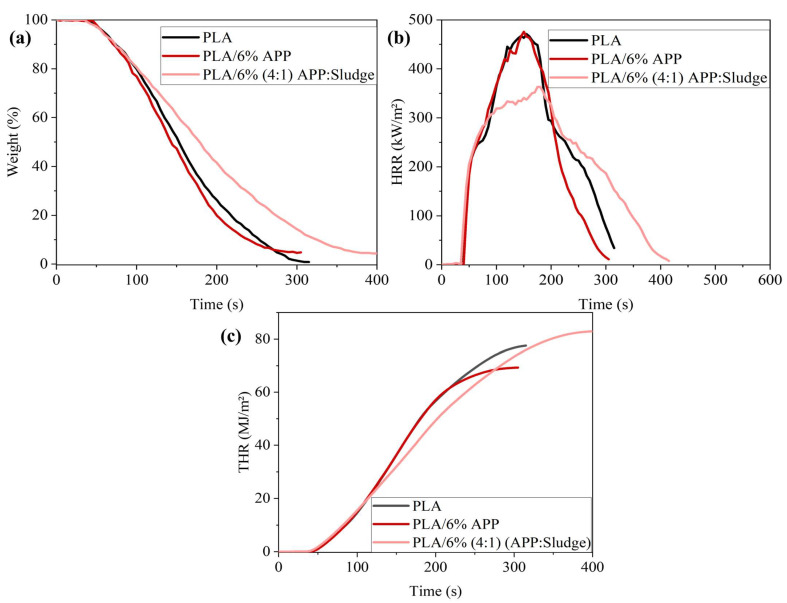
(**a**) Weight, (**b**) HRR and (**c**) THR curves of the PLA/6%(4:1) APP:Sludge.

Figure 10a–c presented the heat release rate (HRR), total heat release (THR), and char residue at 700 °C for PLA and its composites at 6% loading. A summary of the relevant data was provided in Table 4.

PLA ignited at 37 s, displaying a peak heat release rate (pHRR) of 471 kW/m^2^ and a THR of 77 MJ/m^2^, consistent with literature values [56]. PLA/6%APP showed a similar pHRR (476 kW/m^2^) to pure PLA, with a minor reduction in THR and a notable increase in total smoke production (TSP), which was the highest among the tested samples. These results indicate that the addition of 6 wt% APP alone does not significantly improve the flame-retardant performance of PLA. This observation aligns with previous findings where 6 wt% APP was insufficient to notably suppress combustion intensity [54]. In contrast, partial replacement of APP with sludge (4:1 APP:Sludge) resulted in a reduction in the pHRR by 24% compared with PLA/6% APP. TSP was also reduced by 31%, indicating enhanced fire safety and smoke suppression. Although this formulation clearly improves fire performance in terms of pHRR and TSP, it is important to highlight that PLA/6% (4:1) APP:Sludge exhibited a higher THR (83 MJ/m^2^) compared to PLA/6%APP (69 MJ/m^2^). This outcome can be rationalized by considering the relationship between HRR and THR. Since THR corresponds to the integral of the HRR curve over the full combustion process, it depends not only on the maximum HRR but also on the duration of burning. In this case, while PLA and PLA/APP samples extinguished after approximately 300 s, the PLA/APP:Sludge sample sustained a small flame until ~400 s. This extended burning time increased the area under the HRR curve, resulting in a higher THR value.

#### 3.3.3. Char Analysis

**Figure 11:**
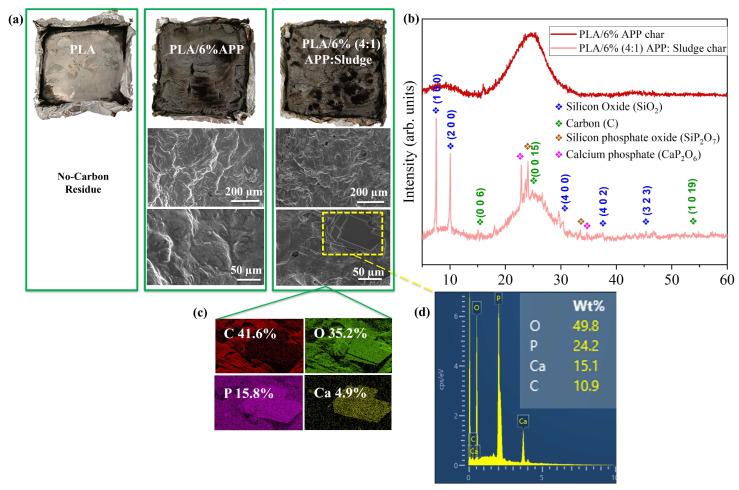
(**a**) Digital images and SEM of char residues, (**b**) XRD spectra for PLA/6% APP and PLA/6% (4:1) APP:Sludge char residues, (**c**) Elemental mapping for PLA/6% (4:1) APP:Sludge char residue and (**d**) Microcrystals EDS spectra.

The char residue of the sample after Cone Calorimeter Test (CCT) was analyzed to gain insights into how the sludge influenced the fire retardancy of PLA. The study included the examination of digital photos and SEM images of the residual material as well as EDS and elemental mapping spectra (Figure 11). As illustrated in this figure, no residue was observed in PLA. However, at a higher magnification for the PLA/6%APP char residue, a typical bulging char characteristic of intumescent materials was observed, devoid of apparent bubbles. In the case of the PLA/6% (4:1) APP:Sludge residue at the same magnification, a similar surface was exhibited, albeit with the appearance of scattered pores (≈10 µm). This indicated that the volatiles emitted during the combustion of the polymer penetrated through the char layer. Raman spectroscopy was also conducted for both residues of PLA/6% APP and PLA/6% (4:1) APP:Sludge (Figure S2), revealing similar I_D_/I_G_ values for both. This suggested that the char did not undergo a loss of quality upon the addition of sludge. At higher magnification (50 μm), distinct differences were evident in the surface morphology of the char residues. The residue from PLA/6% APP retained a structure consistent with observations at 200 μm, characterized by its relatively uniform and amorphous appearance. In contrast, the char residue of PLA/6% (4:1) APP:Sludge exhibited the appearance of microcrystalline structures (Figure 11a), which were absent in the PLA/6% APP residue. The presence of these microcrystals suggested enhanced structural stability and potential contributions to the formation of a protective barrier layer, critical in improving the fire-retardant performance of the composite.

Elemental mapping of the area containing the crystals in Figure 11c revealed the presence of elements such as C, O, P, originated from the phosphorylation and dehydration of APP, as well as minor elements from the sludge. EDS performed only in the formed crystals in Figure 11d indicated a composition predominantly of O (≈50%) and P (≈24%), together with Ca (≈15%) and C (≈11%). This suggests that the crystals are mainly phosphorus and calcium oxides originating from both the sludge and APP during combustion, and they exhibit different morphologies within the char residue (see additional SEM images in Figure S3). Furthermore, in other regions of the char (Figure S4), elements such as Fe and Al were also detected, which can be attributed to their accumulation from the sludge composition prior to combustion.

The formation of these microcrystals in the PLA/6% (4:1) APP:Sludge char residue provided a plausible explanation for the improved fire performance observed when sludge was employed as a synergist. This phenomenon, commonly observed in systems incorporating inorganic fillers, was probably due to the interaction between the acidic components of ammonium polyphosphate (APP) and the inorganic constituents of the sludge at elevated temperatures [57,58]. The resulting crystalline salts acted as effective physical barriers, reducing heat transfer and thereby enhancing the material’s fire safety behavior. Several studies have shown that phosphorus–calcium materials can act as catalytic sites that promote the stabilization of char and alter the release of volatile degradation products, redirecting them towards more oxidized species [59,60,61] thereby reducing the production of incomplete combustion byproducts, as evidenced in CCT results. Divalent or multivalent metallic compounds, including metal oxides, metal sulfates and metal phosphates, have been documented as elements that could facilitate the dehydration and crosslinking among polymer chains and/or fire retardants [46]. This collaborative action was linked to the catalytic potential of these metal compounds in fostering polymer matrix crosslinking alongside APP, encouraging char formation and ultimately enhancing fire resistance.

X-ray diffraction (XRD) analysis was performed on the char residues to confirm the presence of crystalline phases in the PLA/6 % (4:1) APP:Sludge residue. As depicted in Figure 11b, PLA/6% (4:1) APP:Sludge char exhibited various crystalline peaks associated with those observed through SEM visualization. XRD analysis revealed diffraction peaks corresponding to silicon oxide (ICDD: 04-007-2626), graphite (ICDD: 01-074-2329), and calcium phosphate phases (ICDD: 00-015-0204), consistent with the presence of P, Ca, and O elements identified in Figure 11c,d. Additionally, peaks associated with silicon phosphate oxides (ICDD: 00-015-0564) were also detected. PLA/6%APP char did not present any crystalline peaks.

**Figure 12:**
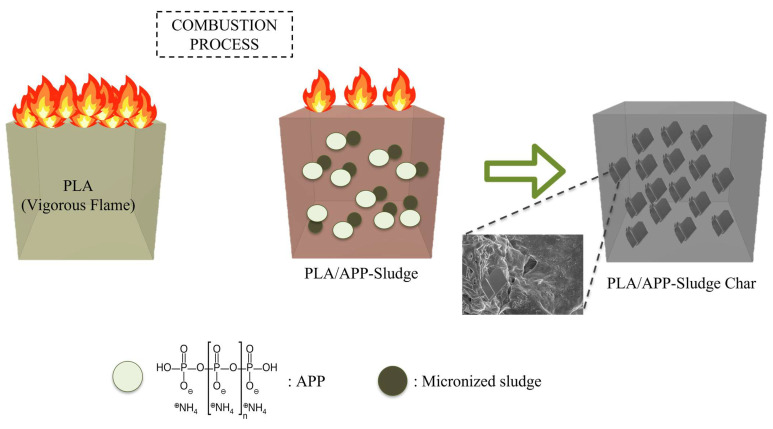
PLA/APP-Sludge combustion process.

The combustion behavior of the samples was illustrated in Figure 12. When exposed to a heat source, pure PLA absorbed thermal energy efficiently and underwent rapid thermal degradation. This resulted in a continuous release of combustible volatiles and elevated heat output, increasing its inherent fire risk. During combustion, chemical interactions between acidic polyphosphoric species from APP and the inorganic components of the sludge promoted the in situ formation of calcium and phosphorus-based crystalline salts. These microcrystals, confirmed by SEM–EDS and XRD analyses, were dispersed throughout the char matrix and were absent in the PLA/APP residue. The presence of these mineral microstructures introduced localized barriers within the char layer, which limited heat transfer and contributed to flame retardancy by reinforcing the structural integrity of the residue. Their formation played a key role in slowing down thermal degradation and flame spread.

### 3.4. TGA-FTIR Analysis

**Figure 13:**
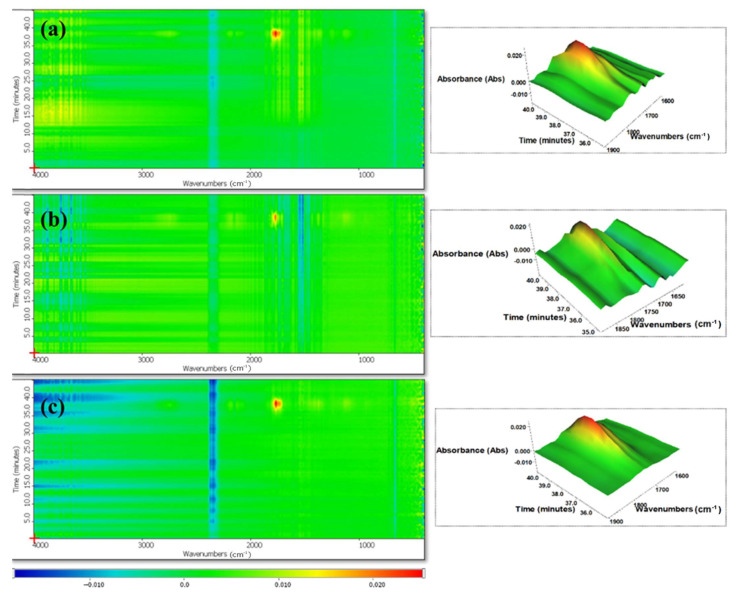
Mapping and absorbance spectra for the volatile compounds at the maximum degradation rate; (**a**) PLA, (**b**) PLA/8% APP and (**c**) PLA/8% (4:1) APP:Sludge.

To further elucidate the pyrolysis behavior and identify the gaseous degradation products during combustion, thermogravimetric analysis coupled with Fourier-transform Infrared spectroscopy (TGA-FTIR) was conducted on PLA composites containing an 8% loading at the peak degradation rate (≈38 min). As depicted in Figure S5, all the spectra displayed characteristic absorption bands corresponding to water (3580 cm^−1^), hydrocarbons (2742 cm^−1^), CO_2_ (2183 cm^−1^), carbonyl compounds (C=O, 1765/1112 cm^−1^), and aromatic compounds (1371 cm^−1^) [20]. These findings indicated that the incorporation of sludge as a synergistic agent did not significantly alter the primary thermal degradation pathways or the composition of gaseous products. Notably, the low absorbance levels observed in all samples (up to 0.020) signified minimal volatile compound emissions, which aligned with the low TSP values obtained from cone calorimeter tests. Among the detected volatile species, carbonyl groups exhibited a prominent peak intensity, as evidenced by the red regions in the volatile compound map (Figure 13). These results underscored the potential of sludge-enhanced PLA composites to maintain favorable fire-retardant properties while emitting reduced quantities of hazardous gaseous byproducts.

### 3.5. Mechanical Properties

**Figure 14:**
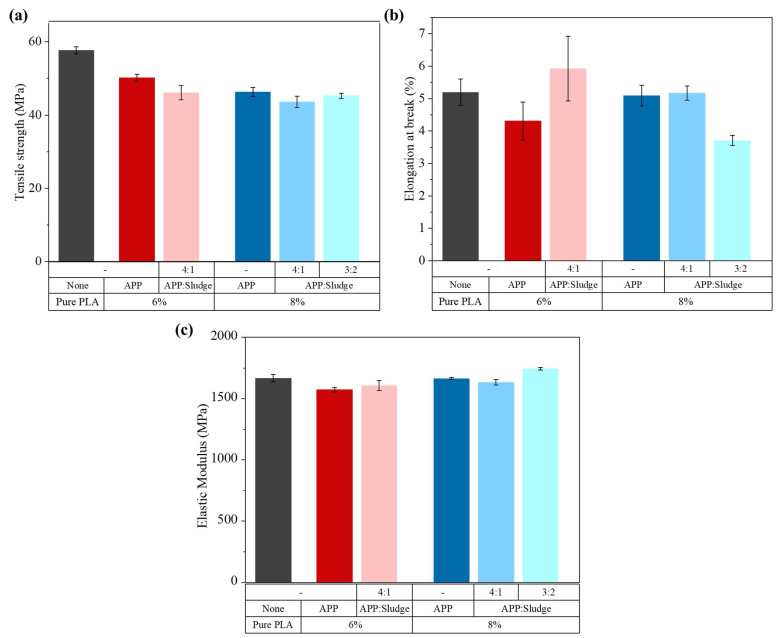
(**a**) Tensile strength, (**b**) Elongation at break and (**c**) Elastic modulus of the PLA and its composites.

Figure 14 summarized the tensile performance of the PLA-based composites. A reduction in tensile strength was observed for all PLA/APP:Sludge formulations relative to pure PLA, consistent with trends commonly reported in polymer composites containing inorganic fillers matrix [53,54]. This mechanical deterioration was attributed to particle agglomeration and poor interfacial adhesion, which induced microvoids and stress concentration points that hindered efficient stress transfer through the matrix. Notably, the tensile strength values of the PLA/8% (3:2) APP:Sludge were very close to that of PLA/8%APP, indicating that at this composition, the partial replacement of APP by sludge did not further compromise mechanical integrity.

Elongation at break of the PLA/6% (4:1) APP:Sludge composite increased by 17% and 43% compared to neat PLA and PLA/6%APP, respectively. This ductility enhancement may be linked to the plasticizing effect of sludge [49,50], which can locally reduce polymer chain packing and increase segmental mobility under tensile load, a trend also observed in rheological analyses. For the elastic modulus, PLA/6% (4:1) APP:Sludge exhibited a higher modulus than PLA/6%APP, though both remained lower than that of neat PLA. This suggested that while the sludge introduced some structural reinforcement due to its mineral content, it also softened the polymer matrix through its plasticizing effect, leading to a trade-off between stiffness and flexibility. In contrast, at 8% loading, PLA/8% (3:2) APP:Sludge achieved the highest modulus among all samples, even surpassing neat PLA. This could be attributed to a higher inorganic phase content and a more efficient filler–matrix interaction, which may promote better stress transfer and restrict chain mobility, resulting in a stiffer material.

This mechanical behavior correlated partially with the degree of crystallinity (Xc) derived from DSC analysis. The PLA/6% (4:1) APP:Sludge sample exhibited the highest crystallinity (Xc = 2.7%) among all formulations, suggesting that sludge may act as a nucleating agent. A slight increase in crystallinity could contribute to stiffness, although in this case, the low overall crystallinity (<3%) confirmed that the dominant factors affecting mechanical performance were filler content, dispersion quality, and matrix–filler interfacial adhesion, rather than crystallinity alone.

### 3.6. Contact Angle Analysis

**Figure 15:**
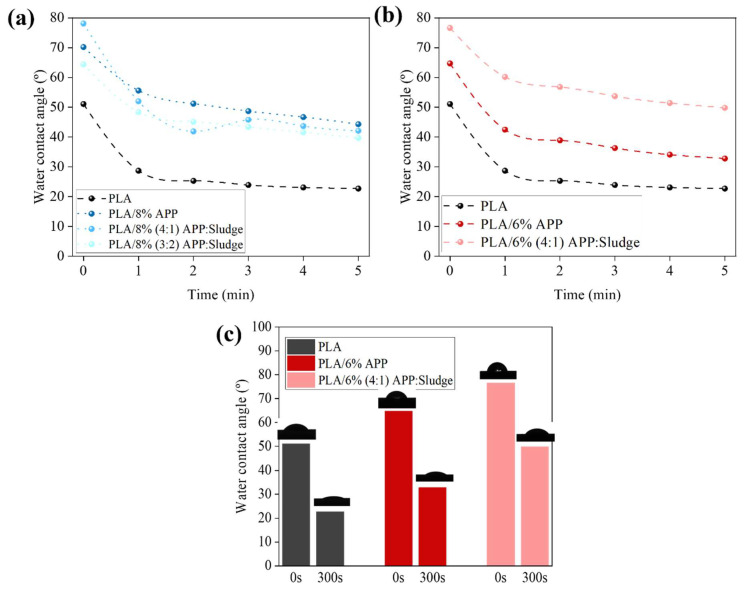
Time dependence of WCA on the PLA composites surfaces for (**a**) 8% and (**b**) 6% loading, (**c**) detailed contact angle for 6% loading at initial time and after 300 s.

As shown in Figure 15, a progressive decrease in water contact angle (WCA) was observed over time, indicating increased water uptake across all samples. Pure PLA exhibited the lowest initial WCA value (51°), reflecting its inherently higher hydrophilicity. In contrast, the PLA/6% (4:1) APP:Sludge composite showed the highest WCA (77°), exceeding even that of PLA/6%APP (65°). This enhancement suggests that the incorporation of sludge contributed to a more hydrophobic surface character, potentially by modifying the surface energy or microstructure of the composite. These findings are consistent with previous studies reporting that the inclusion of inorganic fillers in polymer matrices can reduce water affinity and increase surface hydrophobicity by impeding water diffusion or altering polymer–water interactions [19,62].

### 3.7. Printability

**Figure 16:**
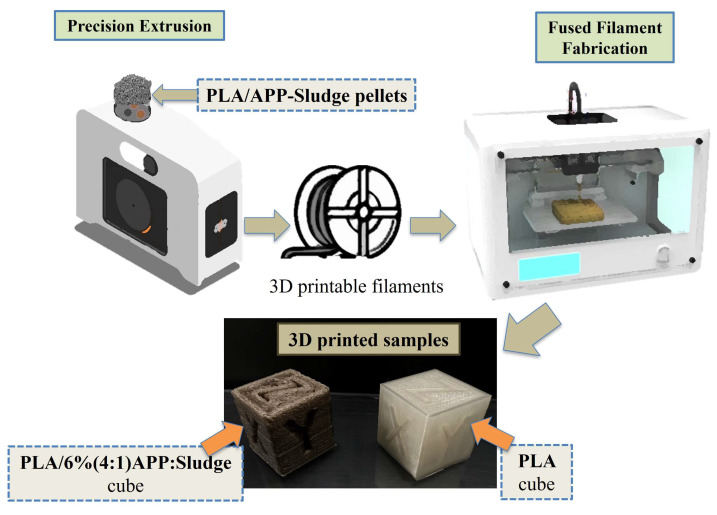
Fabrication process of PLA/APP- Sludge 3D printed samples.

In this section, the printability of the sludge with APP in the manufacturing of a PLA filament will be described. Pellets derived from the extrusion of PLA/6% (4:1) APP:Sludge were fed into a precision extrusion extruder to produce filaments of uniform diameter (See Supplementary Video S4). Subsequently, the filaments were utilized in the fabrication of 3D-printed parts as shown in Figure 16. Optimal 20 × 20 × 20 mm^3^ cubes were successfully obtained using both the material PLA/6% (4:1) APP:Sludge and pure PLA.

Both PLA and PLA/6% (4:1) APP:Sludge printed cubes were exposed to a direct flame for 10 s, and the flammability of the cotton placed underneath was assessed. As observed, the PLA cube underwent combustion across the entire sample, resulting in the dripping of flammable particles. In contrast, the PLA/6% (4:1) APP:Sludge cube was self-extinguished, and no flammable particles were observed, consistent with the UL-94 test results performed on extruded samples (See Supplementary Video S5). This demonstrated the potential of incorporating wastewater sludge as a synergist in PLA-based materials, enabling the production of components with varying dimensions while maintaining fire-retardant properties and reducing APP usage.

## 4. Conclusions

This study demonstrates the potential of anaerobic digester-derived sludge as a sustainable synergistic component in flame-retardant PLA composites. When combined with ammonium polyphosphate (APP), the PLA/Sludge formulations achieved UL-94 V-0 ratings and higher limiting oxygen index (LOI) values compared to PLA/APP alone. Cone calorimetry further confirmed the synergistic effect, with the PLA/6% (4:1) APP:Sludge composite resulting in a nearly 31% reduction in total smoke production (TSP) compared to the PLA/6%APP system and a 24% decrease in peak heat release rate (pHRR) relative to both neat PLA and PLA/APP composites. SEM–EDS of the char revealed phosphorus-and calcium-based microcrystals, formed through interactions between APP and sludge inorganics, which acted as barriers to heat and mass transfer and contributed to improved flame retardancy. From a mechanical perspective, the PLA/6% (4:1) APP:Sludge formulation exhibited greater ductility, with elongation at break increased by 17% and 43% compared to neat PLA and PLA/6% APP, respectively. Moreover, the successful extrusion of filaments suitable for 3D printing highlighted the applicability of these composites. Overall, the integration of sludge as a low-cost, sustainable synergist offers a promising strategy for both improving fire performance and valorizing industrial waste in PLA-based systems.

## Figures and Tables

**Table 1:** Formulations of PLA composites.

Sample	PLA (wt%)	APP (wt%)	Sludge (wt%)
PLA	100	0	0
PLA/8%APP	92	8	0
PLA/8% (3:2) APP:Sludge	92	4.8	3.2
PLA/8% (4:1) APP:Sludge	92	6.4	1.6
PLA/6%APP	94	6.0	0
PLA/6% (4:1) APP:Sludge	94	4.8	1.2

**Table 2:** DSC results of the PLA composites.

Sample	Tg (°C)	Tc (°C)	Tm (°C)	ΔHc − ΔHm (J/g)	Xc (%)
PLA	63.3	118.2	150.9	1.6	1.7
PLA/8% APP	63.5	116.1	151.8	0.9	1.0
PLA/8% (3:2) APP:Sludge	63.2	114.6	151.4	1.6	1.9
PLA/8% (4:1) APP:Sludge	63.4	115.1	152.0	1.9	2.2
PLA/6% APP	63.7	115.8	152.1	1.4	1.6
PLA/6% (4:1) APP:Sludge	63.5	115.0	151.4	2.4	2.7

**Table 3:** UL-94 and LOI results.

		UL-94 (3.2 mm)			
Sample	$\bar{t_{1}+t_{2}}$ (s)	Cotton Ignition (Yes/No)	Dripping (Yes/No)	Rating	LOI (%)
PLA	5	Y	Y (severe)	V-2	22.8
PLA/8%APP	4	N	Y	V-0	31.4
PLA/8% (4:1) APP:Sludge	6	N	Y	V-0	33.0
PLA/8% (3:2) APP:Sludge	2	N	Y	V-0	33.0
PLA/6%APP	3	N	Y	V-0	31.4
PLA/6% (4:1) APP:Sludge	5	N	Y	V-0	33.0
PLA/6% (3:2) APP:Sludge	2	Y	Y	V-2	32.0
PLA/5%APP	11	Y	Y	V-2	30.4

**Table 4:** Cone calorimeter test results.

Sample	TTI (s)	pHRR (kW/m^2^)	THR (MJ/m^2^)	TSP (m^2^)	MARHE (kW/m^2^)	Char Residue @700 °C (wt%)
PLA	37 ± 3	471 ± 7	77 ± 2	0.31 ± 0.1	284 ± 9	0.4 ± 0.2
PLA/6%APP	39 ± 4	476 ± 4	69 ± 1	2.31 ± 0.3	286 ± 6	2.0 ± 0.4
PLA/6% (4:1) APP:Sludge	34 ± 2	363 ± 6	83 ± 1	1.60 ± 0.5	251 ± 7	2.0 ± 0.5

## Data Availability

The original contributions presented in this study are included in the article/supplementary material. Further inquiries can be directed to the corresponding author.

## Supplementary Materials

The following supporting information can be downloaded at: https://www.mdpi.com/article/10.3390/polym17202717/s1. Figure S1. TGA curve for Ammonium Polyphosphate (APP); Figure S2. Deconvolution Raman for PLA/6%APP and PLA/6% (4:1) APP:Sludge char residues; Figure S3. SEM images of the microcrystals (dotted in green squares) formed in PLA/6% (4:1) APP:Sludge; Figure S4. SEM-EDS images of the PLA/6% (4:1) APP:Sludge char; Figure S5. FTIR spectra for the volatile compounds at the maximum degradation stage; Table S1. Sludge Particle size reduction in Horizontal Bead Mill; Table S2. TGA results of the PLA composites; Video S1. UL94_Pure PLA; Video S2. UL94_PLA_8%loading; Video S3. UL94_PLA_6%loading; Video S4. Ignition of 3D printed cubes; Video S5. Precision extrusion fabrication of 3D printed samples.
