# Supplementary material for: Enhancement of Poly(Lactic Acid) Fire Retardancy Through the Incorporation of Sludge Residue as a Synergistic Additive
Source: Polymers (Basel). 2025 Oct 10;17(20):2717. doi: 10.3390/polym17202717 (PMC12567049; doi:10.3390/polym17202717)
Supplement: Supplementary file 1 [file polymers-17-02717-s001.zip › polymers-3891132-supplementary.pdf]

Supplementary Materials

Enhancement of Poly(Lactic Acid) Fire Retardancy Through the Incorporation of Sludge Residue as a Synergistic Additive

Jimena de la Vega[1,2], Antonio Vázquez-López[3] and De-Yi Wang[1,*]

1 IMDEA Materials Institute, C/Eric Kandel, 2, 28906 Madrid, Spain; jimena.vega@imdea.org

2 Department of Materials Science, ETS Ingenieros de Caminos, Polytechnic University of Madrid, 28040 Madrid, Spain

3 Materials Science and Engineering Area, Escuela Superior de Ciencias Experimentales y Tecnología, Universidad Rey Juan Carlos, C/Tulipán s/n, 28933 Madrid, Spain; antonio.vazquez@urjc.es

Correspondence: deyi.wang@imdea.org

Table S1. Sludge Particle size reduction in Horizontal Bead Mill.

		Milling time				
**Speed**	**Sample**	Initial	30min	60min	90 min	120 min
500 rpm	**Average D_{50} (µm)**	56.7	5.9	5.5	5.4	5.10
1000 rpm	**Average D_{50} (µm)**	56.7	4.5	3.9	2.7	2.3
1500 rpm	**Average D_{50} (µm)**	56.7	2.9	2.00	1.2	1.00

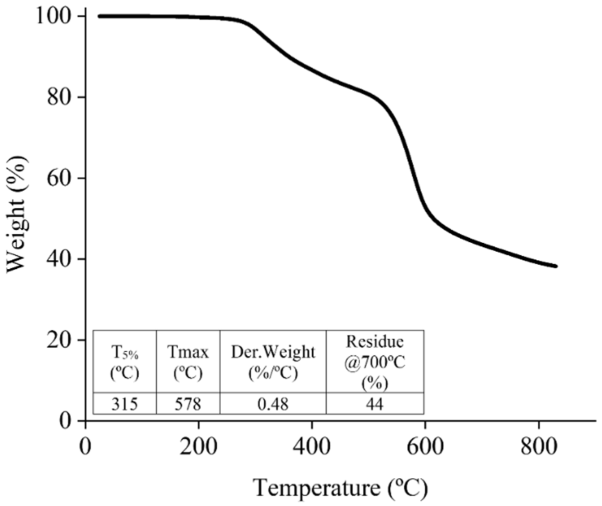

Figure S1. TGA curve for Ammonium Polyphosphate (APP).

According to the TGA results, the thermal decomposition of ammonium polyphosphate (APP) was a three-step process. In the initial step, occurring between 200 and 450 °C, ammonia and

water were released because of the thermal degradation of polyphosphate. The subsequent step, taking place beyond 450 °C, involved the elimination of phosphoric acid, polyphosphoric acid, and metaphosphoric acid through the decomposition of APP [1]. The primary pyrolysis decomposition occurred within the temperature range of 150 to 550 °C. The initial decomposition stage took place between 150 and 380 °C, likely associated with depolymerization reactions. Subsequently, the second stage was around 380–550 °C, marked by the additional degradation of volatile components, as discussed in [2]. Additionally, a minor weight loss was noted at temperatures exceeding 550 °C, indicative of complete decomposition.

Table S2. TGA results of the PLA composites.

**Sample**	$T_{5\%}$ (ºC)	T_{max} (ºC)	**Der.Weight (%/ºC)**	**Yc @700ºC (%)**
PLA	325.9	358.6	4.13	0.0
PLA/8%APP	284.3	354.8	1.67	6.6
PLA/8% (3:2) APP:Sludge	322.6	364.6	2.52	8.2
PLA/8% (4:1) APP:Sludge	320.9	363.9	2.48	7.6
PLA/6%APP	327.4	364.4	2.7	4.9
PLA/6% (4:1) APP:Sludge	324.0	365.3	2.6	4.9

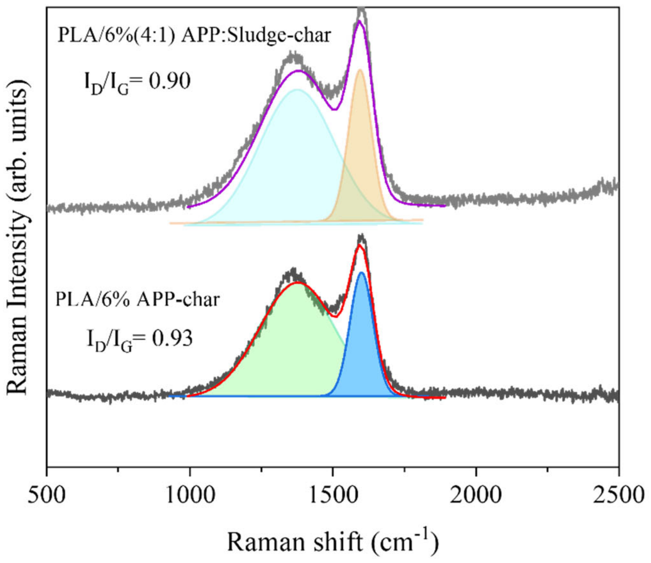

Figure S2. Deconvolution Raman for PLA/6%APP and PLA/6% (4:1) APP:Sludge char residues.

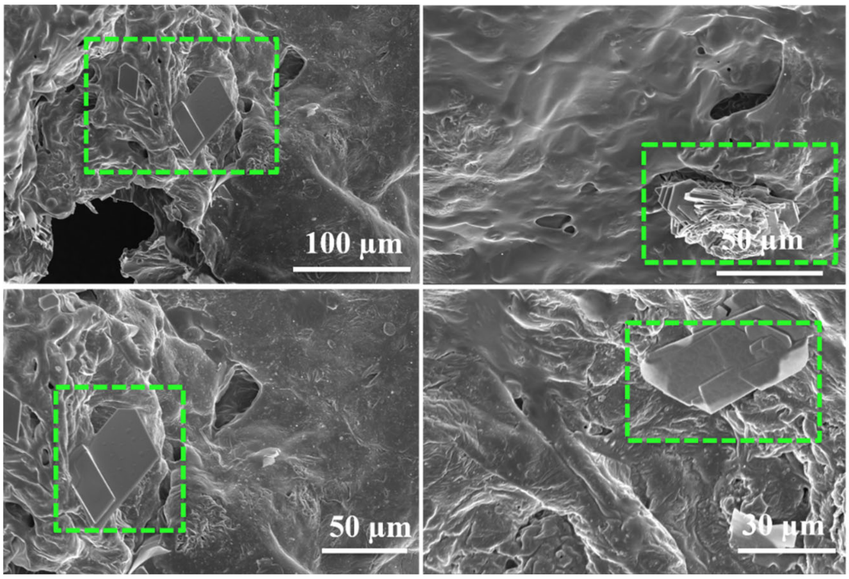

Figure S3. SEM images of the microcrystals (dotted in green squares) formed in PLA/6% (4:1) APP:Sludge.

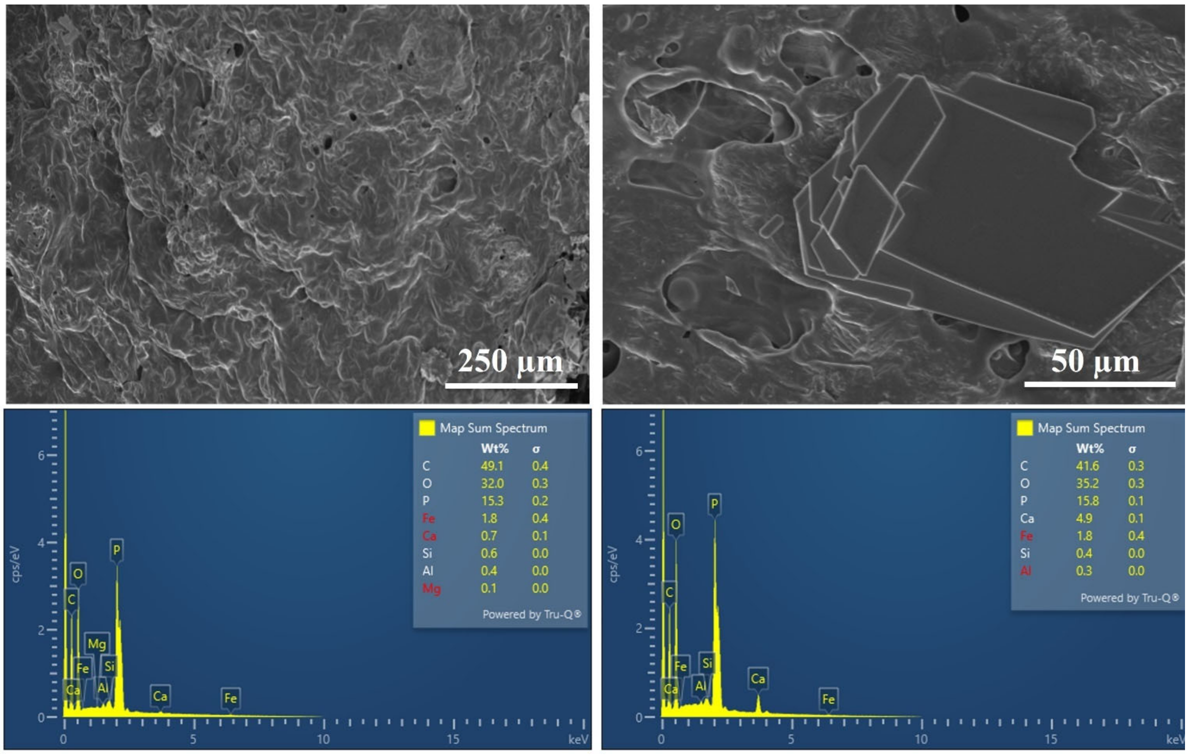

Figure S4. SEM-EDS images of the PLA/6% (4:1) APP:Sludge char.

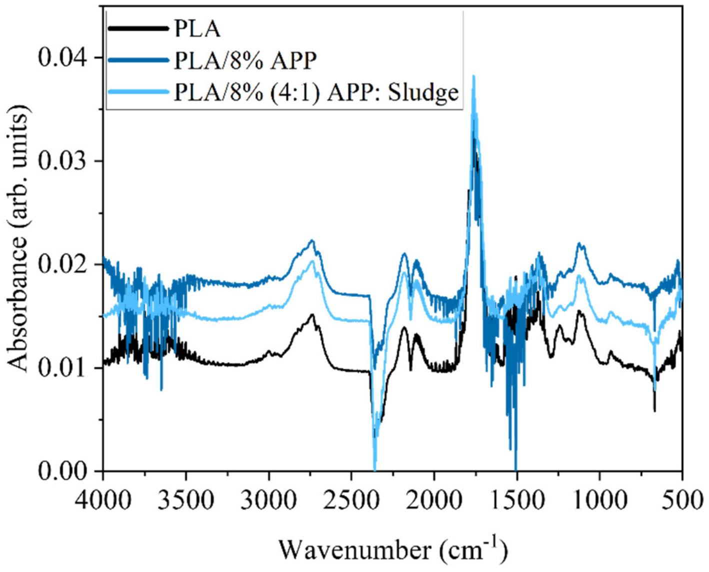

Figure S5. FTIR spectra for the volatile compounds at the maximum degradation stage.

References

1. Cayla, A.; Rault, F.; Giraud, S.; Salaün, F.; Sonnier, R.; Dumazert, L. Influence of Ammonium Polyphosphate/Lignin Ratio on Thermal and Fire Behavior of Biobased Thermoplastic: The Case of Polyamide 11. *Materials* **2019**, *12*, doi:10.3390/ma12071146.
2. Wang, J.S.; Wang, D.Y.; Liu, Y.; Ge, X.G.; Wang, Y.Z. Polyamide-Enhanced Flame Retardancy of Ammonium Polyphosphate on Epoxy Resin. *J Appl Polym Sci* **2008**, *108*, 2644–2653, doi:10.1002/app.27522.
